# The Eukaryotic Linear Motif resource: 2022 release

**DOI:** 10.1093/nar/gkab975

**Published:** 2021-10-29

**Authors:** Manjeet Kumar, Sushama Michael, Jesús Alvarado-Valverde, Bálint Mészáros, Hugo Sámano‐Sánchez, András Zeke, Laszlo Dobson, Tamas Lazar, Mihkel Örd, Anurag Nagpal, Nazanin Farahi, Melanie Käser, Ramya Kraleti, Norman E Davey, Rita Pancsa, Lucía B Chemes, Toby J Gibson

**Affiliations:** Structural and Computational Biology Unit, European Molecular Biology Laboratory, Heidelberg 69117, Germany; Structural and Computational Biology Unit, European Molecular Biology Laboratory, Heidelberg 69117, Germany; Structural and Computational Biology Unit, European Molecular Biology Laboratory, Heidelberg 69117, Germany; Collaboration for joint PhD degree between EMBL and Heidelberg University, Faculty of Biosciences; Structural and Computational Biology Unit, European Molecular Biology Laboratory, Heidelberg 69117, Germany; Structural and Computational Biology Unit, European Molecular Biology Laboratory, Heidelberg 69117, Germany; Zhejiang University School of Medicine, International Campus, Zhejiang University, Haining, China; Biomedical Sciences, Edinburgh Medical School, The University of Edinburgh, Edinburgh, EH8 9JZ, UK; Institute of Enzymology, Research Centre for Natural Sciences, Budapest 1117, Hungary; Structural and Computational Biology Unit, European Molecular Biology Laboratory, Heidelberg 69117, Germany; Institute of Enzymology, Research Centre for Natural Sciences, Budapest 1117, Hungary; VIB-VUB Center for Structural Biology, Vlaams Instituut voor Biotechnologie, Pleinlaan 2, 1050 Brussels, Belgium; Structural Biology Brussels, Department of Bioengineering, Vrije Universiteit Brussel, Pleinlaan 2, 1050 Brussels, Belgium; Institute of Cancer Research, Chester Beatty Laboratories, 237 Fulham Rd, Chelsea, London SW3 6JB, UK; Department of Biological Sciences, BITS Pilani, K. K. Birla Goa campus, Zuarinagar, Goa 403726, India; VIB-VUB Center for Structural Biology, Vlaams Instituut voor Biotechnologie, Pleinlaan 2, 1050 Brussels, Belgium; Structural Biology Brussels, Department of Bioengineering, Vrije Universiteit Brussel, Pleinlaan 2, 1050 Brussels, Belgium; Structural and Computational Biology Unit, European Molecular Biology Laboratory, Heidelberg 69117, Germany; Institute of Pharmacy and Molecular Biotechnology (IPMB), Heidelberg University, Heidelberg, Germany; Structural and Computational Biology Unit, European Molecular Biology Laboratory, Heidelberg 69117, Germany; Justus Liebig University Giessen, Ludwigstraße 23, 35390 Gießen, Germany; Institute of Cancer Research, Chester Beatty Laboratories, 237 Fulham Rd, Chelsea, London SW3 6JB, UK; Structural and Computational Biology Unit, European Molecular Biology Laboratory, Heidelberg 69117, Germany; Institute of Enzymology, Research Centre for Natural Sciences, Budapest 1117, Hungary; Instituto de Investigaciones Biotecnológicas “Dr. Rodolfo A. Ugalde”, IIB-UNSAM, IIBIO-CONICET, Universidad Nacional de San Martín, Av. 25 de Mayo y Francia, CP1650 San Martín, Buenos Aires, Argentina; Structural and Computational Biology Unit, European Molecular Biology Laboratory, Heidelberg 69117, Germany

## Abstract

Almost twenty years after its initial release, the Eukaryotic Linear Motif (ELM) resource remains an invaluable source of information for the study of motif-mediated protein-protein interactions. ELM provides a comprehensive, regularly updated and well-organised repository of manually curated, experimentally validated short linear motifs (SLiMs). An increasing number of SLiM-mediated interactions are discovered each year and keeping the resource up-to-date continues to be a great challenge. In the current update, 30 novel motif classes have been added and five existing classes have undergone major revisions. The update includes 411 new motif instances mostly focused on cell-cycle regulation, control of the actin cytoskeleton, membrane remodelling and vesicle trafficking pathways, liquid-liquid phase separation and integrin signalling. Many of the newly annotated motif-mediated interactions are targets of pathogenic motif mimicry by viral, bacterial or eukaryotic pathogens, providing invaluable insights into the molecular mechanisms underlying infectious diseases. The current ELM release includes 317 motif classes incorporating 3934 individual motif instances manually curated from 3867 scientific publications. ELM is available at: http://elm.eu.org.

## INTRODUCTION

Short linear motifs (SLiMs) are a distinct class of protein functional modules that participate in protein-protein interactions and act as sites of post-translational modification (PTM). The defining feature of SLiMs is their compact interfaces encoded in short linear stretches (commonly 3–15 residues long) of the protein sequence and the lack of a requirement for stable tertiary structure for their function, though many motifs fold upon binding. Most SLiMs are found within the structurally flexible and accessible intrinsically disordered regions (IDRs) of a proteome and are hallmarked by their evolutionary conservation in these rapidly evolving regions (1). While specific positions in a motif region are evolutionarily conserved, marking the key specificity and affinity determinants of the motif, a lower degree of sequence conservation can often be observed in the flanks. However, these regions can still contribute to binding by fine tuning the generally weak interaction affinity (typically low micromolar) of these regions. Moreover, SLiM-mediated interactions are often cooperative with multiple motifs contributing to a given binding event, such that these interfaces provide strong yet dynamic interactions (2).

SLiMs are core components across numerous cellular processes such as replication, differentiation and apoptosis. Their regulatory importance is highlighted by their role in vital cellular pathways, including cell cycle, endocytosis, cytoskeleton dynamics and intracellular signal transduction. The role of SLiMs in protein trafficking, post-translational modification and protein degradation underpins robust signalling regulation and contributes to spatiotemporal and contextual control of the signalling output (3). Mutations in the sequence regions containing SLiMs can contribute to disease states such as cancer (4,5).

Diverse eukaryotic, bacterial and viral pathogens mimic SLiMs present in host cell proteins to hijack cellular signalling to their advantage as part of the infectious cycle (6,7). The hijacking of SLiMs via pathogens can target varied cellular components which involve integrin signalling, endocytic and trafficking pathways and the cytoskeleton machinery (actin and microtubule dynamics). SARS-CoV-2, the coronavirus culpable for the COVID-19 pandemic, has already been shown to use SLiM mimics on the Spike protein to interact with host cell receptors to facilitate viral entry into cells (8,9). Given their relevance in health and disease, SLiM-mediated interactions are increasingly being pursued as targets for therapeutic intervention (5,7,8,10,11).

Considering the importance of SLiMs in cell regulation, the Eukaryotic Linear Motif (ELM) resource was created to scrutinise and systematically capture motif information from the literature. For nearly two decades, ELM has shared high-quality manually curated motif data with the community and has matured as the most widely used motif biology knowledgebase. Availability of these curated data provides avenues to discover novel SLiMs alongside serving as training data in developing bioinformatics toolkits and analysis workflows. In addition, the ELM web server allows users to search their proteins of interest to find candidate SLiMs that experimentalists can probe to test their role in cellular systems.

In this paper, we report on the growth of the ELM dataset since our previous publication (12).

## THE ELM RESOURCE

ELM (Eukaryotic Linear Motif) is a freely accessible resource for understanding and exploring the biology of SLiMs. At the core of the resource is a manually curated set of motif instances derived from the experimental literature (12,13). Individual ELM motif instances that share a biological function, binding partner or recognition features are grouped into an ELM motif class. A motif class summarises the motif function and its contextual knowledge, such as the cellular location of the proteins bearing motif instances and the interacting partner domains. Each class has a manually created motif pattern defining the key specificity and affinity determinants of the ELM motif class. The motif pattern is represented by a standard POSIX regular expression (https://en.wikipedia.org/wiki/Regular_expression and see the ‘Regular expressions’ section at http://elm.eu.org/infos/help.html for a detailed description of ELM consensus definitions) and derived from the evolutionary and structural information of the motif instances. ELM motif classes are further classified based on their high-level function into six broad categories: cleavage (CLV), degradation (DEG), docking (DOC), ligand (LIG), modification (MOD) and targeting (TRG) motifs.

Periodically, existing classes may be updated as new information becomes available. The resource stores the annotated data in a PostgreSQL relational database [http://www.postgresql.org/]. The data in the backend is accessed via the Django web framework [https://djangoproject.com/] and is served on the front end web interface designed for an intuitive and user-friendly browsing experience. The ELM resource also provides access to the functional site prediction toolkit, enabling users to search candidate motifs within their proteins of interest. Based on sequence matches to the motif regular expressions, the detected candidate motifs are mapped and shown onto the protein sequence. Furthermore, distinction between plausible true and false positive candidate motifs is made with logical filters based on globular domain, structure and contextual knowledge.

The data stored in the ELM resource is freely accessible and downloadable in a range of formats. Details of the available formats and datasets can be found at http://elm.eu.org/downloads.html. In addition, a REST-API is available to programmatically search ELM class consensus matches against proteins of interest. The usage details of the motif search REST-API service are available at http://elm.eu.org/api/manual.html.

## DATA UPDATES

**Figure 1.**
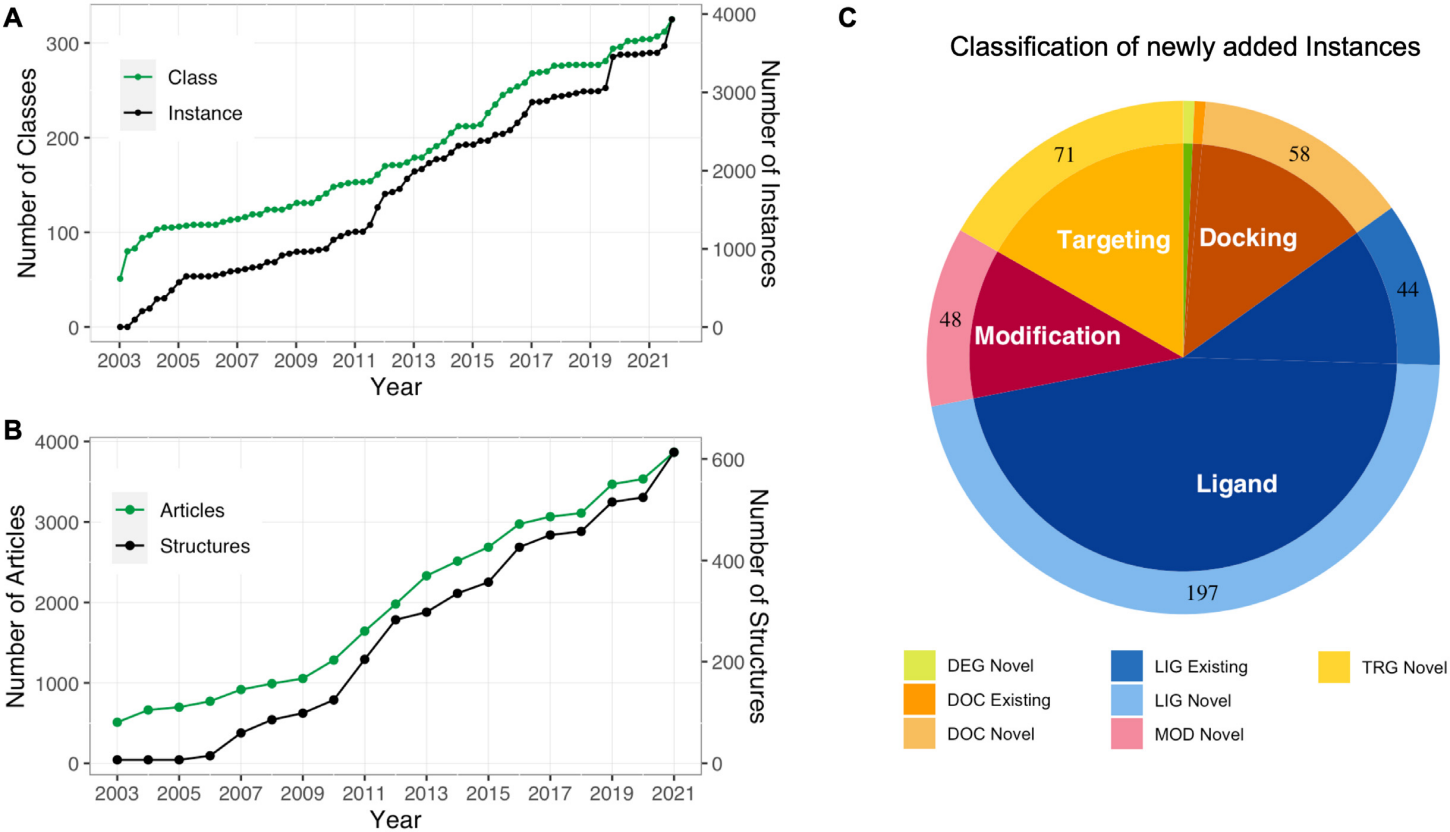
Data growth progression in the ELM resource. (**A**) Cumulative number of annotated motif classes and instances added in ELM. (**B**) Cumulative number of structures and papers added to the database. (C) Distribution of newly added instances between novel and already existing motif classes (lighter and darker shades in the outer circle, respectively) belonging to five different functional categories. The pie chart only includes the 35 classes where new instances have been added: for visual clarity, classes with less than five instance additions are not labelled in the pie-chart. The plotted instances in the pie-chart also include remapped instances from previously existing classes in the database. The plots were produced using ggplot2 in R-studio (http://www.rstudio.com/).

Since the last release in 2020 (12), ELM curation has focused especially on linear motifs regulating the cell cycle, cytoskeleton and vesicle trafficking, regulation of cellular phase separation, and pathogen hijacking. ELM currently contains motif-centric biological information for 317 motif classes (Figure 1A; Table 1), of which 30 have been added in the current update (Table 2). Similarly, the number of annotated motif instances has expanded to 3934 (Figure 1A; Table 1) including 411 newly added instances (Figure 1C). Several existing motif classes and instances have been updated and revised to capture recent advances in the literature (Table 2), while two were made obsolete (due to being replaced by six new, better defined variant classes). 3867 scientific publications are recorded in the ELM resource (Figure 1B). The current ELM release also includes 98 additional structures of motif instances, most of which are bound to their motif binding partner. The resource now contains 616 structures cross-referenced to the RCSB-PDB (14) and PDBe (15) databases (Figure 1B). Furthermore, we have updated the Kyoto Encyclopedia of Genes and Genomes (KEGG) (16) pathway mapping for ELM instances, with the current ELM release possessing 1063 KEGG pathway links mapped on 1297 motif-containing proteins. In addition, ELM data capture 2394 interactions between ELM instances and their motif-binding interaction partners. The binding affinities have been curated for 571 of these motif-mediated interactions. More details on the nature of ELM data are presented in Table 1.

**Table 1.** Concise summary of data captured in the ELM resource

Functional sites		ELM classes		ELM instances		GO terms		PDB structures	PubMed links
Total	190	317		3934		884		616	3867
By Category		LIG	178	Human	2222	Biological Process	489		
		MOD	38	Mouse	373				
		DOC	38	Rat	171	Cellular Component	184		
		DEG	26	Yeast	330				
		TRG	26	Viruses	277	Molecular Function	211		
		CLV	11	Others	561				

**Table 2.** New and revised ELM entries in the current ELM release

New ELM classes		
ELM class identifier	#Instances	ELM class (short) description
DEG_SCF_FBXO31_1	3	The C-terminal degron of cyclin D proteins is bound by the FBXO31 F-box E3 ligase of the SCF (Skp1-Cullin-Fbox) complex.
DOC_CDC14_PxL_1	10	The PxL substrate docking motif enhances the Cdc14 phosphatase–substrate interaction and promotes subsequent dephosphorylation.
DOC_CYCLIN_D_Helix_1	3	The Cyclin D Helical docking motif mediates binding of substrates to a site on Cyclin D different from the hydrophobic pocket and enhances substrate phosphorylation by CyclinD/Cdk4-6 complexes.
DOC_CYCLIN_yCln2_LP_2	18	The budding yeast G1/S cyclins Cln1 and 2 bind a specific leucine- and proline-rich (LP) docking motif on G1-specific target proteins.
DOC_CYCLIN_yClb3_PxF_3	4	The hydrophobic patch (hp) of the G2 phase cyclin from budding yeast, Clb3, binds a specific PxF docking motif on regulators and target proteins.
DOC_CYCLIN_yClb1_LxF_4	13	The LxF motif found in budding yeasts serves as a docking site for mitotic cyclin-CDK complexes (M-CDK). It is found in both regulators and mitotic phosphorylation target proteins.
DOC_CYCLIN_yClb5_NLxxxL_5	5	Cyclin hydrophobic patch docking motif NLxxxL specific for S-phase cyclins Clb5 and Clb6 in budding yeasts.
DOC_MIT_MIM_1	5	C-terminal LxxR[FL]xxL based type 1 MIT interacting motif (MIM1) that docks at the MIT domain present in some ESCRT-III proteins.
LIG_ActinCP_CPI_1	15	The conserved capping protein interaction (CPI) motif is employed by a diverse set of proteins to allosterically down-regulate actin filament capping by CP and thereby fine-tune actin assembly dynamics.
LIG_ActinCP_TwfCPI_2	4	The highly conserved twinfilin-type actin capping protein interaction (CPI) motif is employed by twinfilins to maintain the dynamic actin capping/decapping cycles of CP and to counterbalance the effects of negative regulators.
LIG_DLG_GKlike_1	14	The guanylate kinase-like domain of DLG family membrane-associated scaffolding proteins binds phosphorylated motifs in SAPAPs and other protein partners.
LIG_Integrin_RGD_TGFB_3	5	A C-terminally extended subtype of the canonical RGD motif strongly binding to integrins αvβ6 and αvβ8.
LIG_Integrin_RGDW_4	18	A C-terminally extended subtype of the canonical RGD motif strongly binding to integrins αIIbβ3 and αvβ3.
LIG_Integrin_KxxGD_FGGC_5	5	An αIIbβ3 integrin-specific, C-terminal variant of the RGD motif where a displaced lysine substitutes for the canonical arginine.
LIG_KLC1_Yacidic_2	3	A kinesin cargo motif binding to the TPR domain of KLC1 found in JIP1 and TorsinA.
LIG_LSD1_SNAG_1	11	A repressor motif found in some zinc finger transcription factors binds to the amine oxidase domain of LSD1.
LIG_LYPXL_yS_3	2	The yeast short version of the LYPxL motif binds the V-domain of Bro1 and Rim20, proteins involved in endosomal sorting and pH signalling.
LIG_LYPXL_SIV_4	3	The SIV helical version of the LYPxL motif binds the V-domain of Alix, a protein involved in endosomal sorting.
LIG_NRP_CendR_1	12	The CendR motif has a carboxy-terminal arginine, which binds to the Neuropilin b1 domain binding site. CendR motifs are either located at the protein C-terminus or are generated by internal cleavage by a polybasic protease, such as Furin
LIG_PCNA_TLS_4	3	The PCNA binding motifs include the PIP Box, PIP degron, the APIM and the TLS motif. These motifs are found in proteins involved in DNA replication, repair, methylation and cell cycle control.
LIG_RuBisCO_WRxxL_1	20	The WRxxL RuBisCO-binding motif present in Pyrenoid proteins promotes the assembly of this algal organelle and its different compartments.
LIG_SH3_CIN85_PxpxPR_1	60	The non-canonical SH3-binding motif is recognized primarily by adaptor proteins CIN85 and CD2AP, which are involved in RTK regulation, endocytosis, lysosomal degradation, actin cytoskeleton dynamics regulation, and signal transduction
LIG_WRC_WIRS_1	22	WRC interacting receptor sequence (WIRS) is a highly conserved and widespread interaction motif that is employed by diverse membrane proteins to recruit the WRC to initiate the dynamic rearrangements of the actin cytoskeleton.
MOD_CDC14_SPxK_1	48	A subset of Cdk phosphorylation sites conform to the (S)PxK/r pattern that serves as an optimal Cdc14 dephosphorylation site, allowing high catalytic efficiency.
TRG_DiLeu_BaEn_1	23	Classical adaptin sigma subunit-binding acidic dileucine motifs sorting in Endosomal-Basolateral trafficking.
TRG_DiLeu_BaEn_2	4	Phe-containing variant adaptin sigma subunit-binding acidic dileucine motifs sorting in Endosomal-Basolateral trafficking
TRG_DiLeu_BaEn_3	4	Diglutamate-containing variant Adaptin sigma subunit-binding acidic dileucine motifs sorting in Endosomal-Basolateral trafficking.
TRG_DiLeu_BaEn_4	4	Acidic dileucine motifs with a monoleucine preference and extra glutamate sorting in Endosomal-Basolateral trafficking.
TRG_DiLeu_LyEn_5	18	Dileucine motifs lacking Glu + 1 with Pro-Arg preference at + 4 sorting in Endosomal-Basolateral-Lysosomal trafficking.
TRG_DiLeu_BaLyEn_6	18	Acidic dileucine motifs with Arg or Pro preference at position 4 interacting with AP-3 and sorting in Endosomal-Basolateral-Lysosomal trafficking.
Extensively revised ELM classes		
DOC_CYCLIN_RxL_1	31	Both fungal and mammalian S-phase Cyclin/CDK complexes recognize specific RxL docking motifs in their target proteins.
LIG_LIR_Gen_1	54	Canonical LIR motif that binds to Atg8/LC3 protein family members to mediate processes involved in autophagy.
LIG_LIR_Nem_3	11	Nematode-specific variant of the canonical LIR motif that binds to Atg8 protein family members to mediate processes involved in autophagy.
LIG_LYPXL_S_1	18	The short version of the LYPxL motif binds the V-domain of Alix, a protein involved in endosomal sorting.
LIG_LYPXL_L_2	4	The long version of the LYPxL motif binds the V-domain of Alix, a protein involved in endosomal sorting.

## CHALLENGES IN MOTIF ANNOTATION, ASSIGNMENT AND QUALITY ASSESSMENT

While drafting motif entries for ELM, several issues can make the task daunting and hamper the annotation process. One common problem is the lack of a clear identity for the protein being studied in the paper undergoing annotation. These issues are often related to the use of an ambiguous gene or protein name, or the omission of the source species. Other complicating scenarios appear when multiple potential matches for a motif or motif-binding domain are present in a protein and the paper's text does not clearly state which of the modules are being experimentally tested. In these cases it is sometimes not possible to annotate the motif instance or define the interacting partner domain region.

Another commonly observed case deals with the mutagenesis of a predicted motif within the folded region of a protein. Such mutations will often partially or fully unfold a protein thereby altering protein function. As a result, the outcome from such a study will be misleading and will not help identify the actual functional motif region. In some cases the mutant phenotype can even match the expected phenotype for a motif mutation. For example, the mutagenesis of a predicted targeting motif buried in a folded region could result in protein unfolding and subsequent mislocalisation. The motif data is still extracted from such papers, and if they match the regular expression of the motif, they are assigned as false positives in ELM. In some articles, the experimental approaches applied do not conclusively prove the existence of a motif; for example, neither phenotypic or other complex functional assays nor simple peptide pull-downs from cell extracts unambiguously demonstrate the existence of SLiMs. These types of approach need to be complemented by more direct biophysical experiments.

The ELM annotation workflow ensures quality assessment of the motifs by applying various measures. These involve assessing motifs for evolutionary conservation, surface accessibility, and inspecting structures for specificity determining residues, among other evaluations (17). Annotators execute homology searches against biological databases to gather input sequences for building multiple sequence alignments. These alignments are inspected by eye and used to gauge the evolution of motif-containing sequence stretches, which helps define the motif regular expression and its taxonomic distribution. Since SLiMs are mainly present in intrinsically disordered regions, we evaluate motif accessibility with disorder prediction tools and use structural information as available. Having access to protein structures with both motif and interacting partner helps even further as the annotator can inspect a protein complex to define the specificity determinant positions and motif boundaries with more confidence. Further, SLiMs can have variable sequence regions in their flanks to fine-tune the specificity. The handiness of structural data makes it simpler to capture such residues in the motif definition, resulting in a more stringent motif definition and fewer false-positive matches. When structures of the motif in the complex are absent or have lower resolution, annotators put extra effort into preparing high-quality sequence alignments and find more supporting biological context and literature evidence to assign the motif key positions.

## SLiMs IN CELLULAR SYSTEMS/AREAS COVERED IN THE CURRENT ELM UPDATE

### Cell cycle regulation by cyclin:Cdk and phosphatase complexes

Cell division is the universal process through which cells duplicate their genetic material and undergo cytokinesis to produce two daughter cells. While the details vary across eukaryotes, one major biochemical switch, the cyclin:cyclin dependent kinase (cyclin:Cdk) complex, controls cell cycle progression from yeast to mammals through the timed orchestration of substrate phosphorylation (18). Cell cycle progression is driven by a precisely timed oscillation in the activity of cyclin:Cdk complexes that marks the passage through the G1, S, G2 and M cell cycle phases. SLiMs play a major role in cell cycle regulation by controlling the docking of substrates and regulators to cyclin:Cdk and phosphatase holoenzymes, and also by regulating substrate stability and subcellular localisation (18).

**Figure 2.**
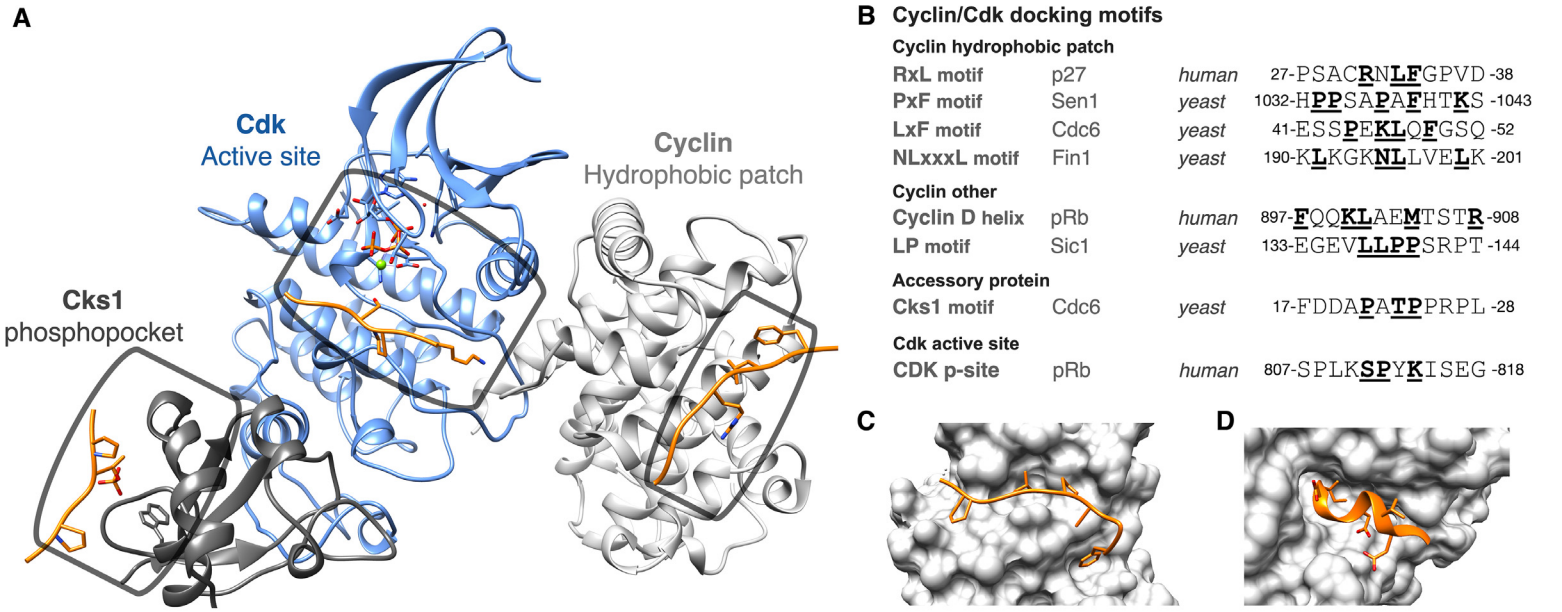
Structural information on motif-mediated regulation by and of the Cyclin-dependent kinases. (**A**) Representative structure of the cyclin:Cdk:Cks holoenzyme showing the three structurally characterised motif-recognising regions of the complex: two docking pockets on the cyclin and CKS subunits and the active site of the holoenzyme on the Cdk subunit. The image is constructed from two structures: a crystal structure of *human* Cdk2:Cyclin A2 in complex with a peptide containing both the SP phosphosite substrate and RxL docking motif of *human* Cdc6 [PDB ID:2CCI], and a crystal structure of a *yeast* Cdc6 phosphopeptide in complex with *yeast* Cks1 [PDB ID:4LPA]. Cks1 was placed on the holoenzyme based on the Cks2:Cdk1 interface in a Cdk:cyclin B1:Cks2 structure [PDB ID:4YC3]. (**B**) Table of the diverse motifs that allow docking and modification by the cyclin:Cdk:Cks holoenzyme split by their pocket on the the complex. Bold and underlined residues are the defined residues from the ELM class for the motif. (**C**) Close-up structure of the interface of *yeast* dual specificity-protein phosphatase CDC14 (grey) in complex with the PxL Cdc14 docking motif of *yeast* SIC1 (orange) [PDB ID:6G86]. (**D**) Close-up of the structure of the interface of *human* F-box only protein 31 (FBXO31) (grey) in complex with the FBXO31 degron of *human* cyclin D1 (orange) [PDB ID:5VZU].

SLiMs mediate substrate docking to the cyclin and Cks1 regulatory subunits of the cyclin:Cdk:Cks1 holoenzyme and their phosphorylation by the Cdk catalytic subunit (Figure 2A, B). In this ELM update, we defined multiple SLiMs that control cyclin and phosphatase docking and cyclin degradation. This effort comprises 8 of the 30 new motif classes in ELM, 104 new motif instances and the revision of an existing motif class (Table 2). Cyclin-docking motifs determine the specificity of substrate phosphorylation at specific cell cycle stages (19). The canonical cyclin-docking RxL motif, DOC_CYCLIN_RxL_1, mediates substrate docking to the hydrophobic patch (*hp*) of yeast and mammalian cyclins (20,21). In the current update, five new motifs mediating cyclin-specific docking have been added (Figure 2B). In budding yeasts, the divergence of the *hp* gave rise to a family of related RxL-like docking motifs (NLxxxL, PxF and LxF). The NLxxxL motif, DOC_CYCLIN_yClb5_NLxxxL_5, mediates substrate docking to S-phase cyclins Clb5/6 (22). The LxF motif, DOC_CYCLIN_yClb1_LxF_4, confers preferential binding of substrates and inhibitors to M-phase cyclins Clb1/2 (23). The PxF motif, DOC_CYCLIN_yClb3_PxF_3, confers binding to G2-phase cyclin Clb3 (24). Docking to G1-cyclins is mediated by motifs that target a surface distinct from the *hp*. The LP motif, DOC_CYCLIN_yCln2_LP_2, mediates binding to late G1-cyclins Cln1/2 and Ccn1 in yeasts (25–27) and is conserved through the fungal lineage (28). In mammals, docking of retinoblastoma-family proteins Rb, p107 and p130 to Cyclin D:Cdk4/6 complexes is mediated by a helical motif, DOC_CYCLIN_D_Helix_1, that cooperates with the RxL and LxCxE motifs to phosphorylate Rb-family proteins early in G1, a key step required for the G1 to S phase transition (29).

The phosphorylation activity of cyclin:Cdk complexes is counteracted by opposing phosphatase activity and regulated cyclin degradation. The Cdc14 dual-specificity phosphatase is required for mitotic exit in budding yeasts (30) by triggering the dephosphorylation of key Cdk1 substrates (31). The PxL docking motif DOC_CDC14_PxL_1 mediates the binding of substrates to the non-catalytic N-terminal domain of the yeast Cdc14 homodimer (32), enhancing target recognition and dephosphorylation (Figure 2C). The newly annotated MOD_CDC14_SPxK_1 motif mediates recognition of specific phosphoserine residues by the C-terminal catalytic domain of Cdc14. Cdc14 strongly favours dephosphorylation of phosphoserines followed by a proline, with an additional positively charged residue downstream (SPxK/R) which matches the Cdk serine phosphorylation site specificity (33). The current release also includes DEG_SCF_FBXO31_1, a phospho-independent degron that mediates Cyclin D protein degradation through binding to the SCF-FBXO31 E3 ligase (34,35) (Figure 2D). Mutation of these degron sequences and the flanking regions regulating nuclear export can contribute to cyclin D overexpression observed in human tumours (36).

### Cytoskeleton and vesicle trafficking

Precise control over actin filament polymerisation is essential for eukaryotic cells and SLiMs play key roles in the related regulatory mechanisms. The current release of ELM includes three new motif classes that directly influence actin filament growth. Two of these, the ‘canonical’ capping protein-interaction (CPI) motif (LIG_ActinCP_CPI_1) and the twinfilin-type CPI motif (LIG_ActinCP_TwfCPI_2), affect actin polymerisation through direct binding of the heterodimeric actin capping protein (CP) that binds to and limits the polymerisation of the barbed ends of actin filaments. While binding of regulators through the canonical CPI motif allosterically down-regulates the capping activity of CP (37), the binding of twinfilins maintains its dynamic capping/de-capping exchange cycle and protects it from negative regulators (38) (39). The third newly entered motif influencing actin filament growth is the conserved WAVE regulatory complex (WRC)-interacting receptor sequence (WIRS; LIG_WRC_WIRS_1) that is employed by a diverse group of membrane proteins to recruit the WRC to initiate rearrangements of the actin cytoskeleton (40).

**Figure 3.**
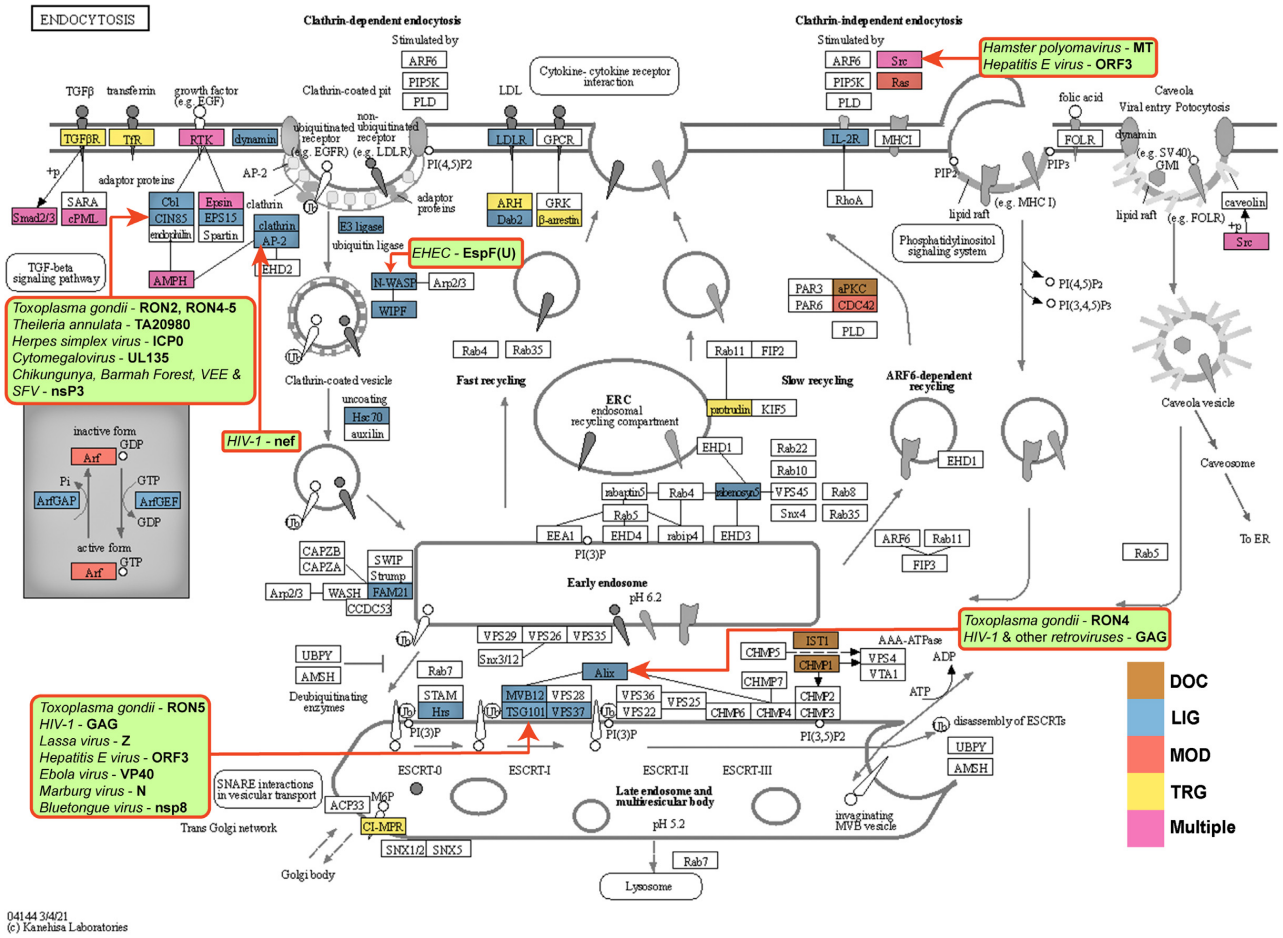
SLiM-mediated interactions in the endocytosis network (KEGG:hsa04144). Proteins with ELM instance annotations are coloured according to motif class: docking—light brown; ligand binding—light blue; modification—salmon; targeting—yellow; multiple classes—pink. Pathogens and their proteins hijacking the endocytosis pathway components are overlaid on the network (light green background rectangles with rounded corners). A red arrow from the rectangle points to the hijacked protein in the network. See ELM for more details such as the binding domains being hijacked by the pathogens. Reproduced from https://www.kegg.jp/pathway/hsa04144 with permission.

SLiMs play prominent roles in regulated transport of cargoes between cell compartments by membrane vesicles and the maintenance of complex endomembrane systems (Figure 3). The ESCRT (endosomal sorting complex required for transport) machinery performs membrane remodelling, sorting and scission events. A number of ESCRT complex proteins contain microtubule interacting and trafficking (MIT) domains that provide distinct docking surfaces for different MIT-interacting motifs (MIMs) found in various ESCRT-III subunits. MIM1 (DOC_MIT_MIM_1) is an α-helical MIM that regulates the delay of cytokinetic abscission, turnover and endosomal sorting of ESCRT-III proteins (41).

LYPxL motifs bind the V-domain of Alix, another ESCRT system component involved in endosomal sorting of membrane proteins. Although our insights into the cellular roles of these motifs are rather limited, they are of prime importance because several viruses use them to hijack the ESCRT machinery for the budding of viral particles from the host cell membrane (42). In the current release we revised two previously annotated subtypes of the motif that mainly rely on viral motif instances (short: LIG_LYPXL_S_1 and long: LIG_LYPXL_L_2) and introduced two novel subtypes, yeast-specific (LIG_LYPXL_yS_3) and long helical SIV (LIG_LYPXL_SIV_4).

This release also includes the classical adaptin-binding acidic dileucine motif (TRG_DiLeu_BaEn_1) and its five variants that almost exclusively occur on the cytosolic side of transmembrane proteins, frequently located near to the N- or C-termini. These motifs bind to a highly conserved site on the sigma subunits of clathrin-associated adaptin complexes (adaptins AP1-4) to initiate clathrin-mediated endocytosis or protein sorting to endosomes/lysosomes (43,44). Dileucine motif-mediated interactions are often hijacked by pathogens, for instance by the Nef protein of HIV (45).

**Figure 4.**
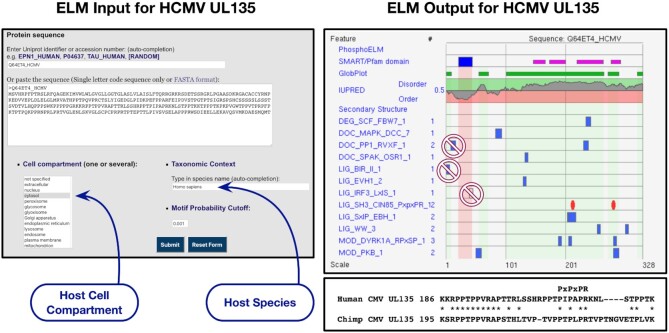
Setting up the ELM server correctly to query viral proteins for SLiM candidates. The example uses IDP-rich UL135 transmembrane protein of Cytomegalovirus for which the natural host is human. UL135 has an N-terminal transmembrane helix and the following sequence faces into the host cell and therefore the correct cell compartment to use is cytosol. The correct species is the host *Homo sapiens*. For the illustration, the motif probability score has been set stringently (0.001) to exclude many matches to motifs with low sequence complexity. In the output, the two recently added PxpxPR motif instances (50) are shown as red ovals. All other motif matches are hypothetical. Three motif matches have conflicting compartment information and are therefore highly improbable. One of the PxpxPR motifs is conserved in a related Cytomegalovirus which infects Chimpanzee (Lower right box).

CIN85 and CD2AP are two large signal integrator adaptor proteins functioning in receptor tyrosine kinase (RTK) signalling and downregulation, endocytosis and cytoskeletal rearrangements, and the clustering and signalling of various other membrane receptors (46). Numerous interaction partners of the two proteins employ PxpxPR motifs (LIG_SH3_CIN85_PxpxPR_1) to bind to one or more of their SH3 domains, including their major interactor, the E3 ligase CBL. The central role of this motif in cell regulation is well illustrated by the variety of viral (several alpha viruses (47), Herpes simplex virus (48), Hepatitis C virus (49) and Human cytomegalovirus (Figures 3 and 4) (50)) and eukaryotic pathogens (*Theileria annulata* (51) and *Toxoplasma gondii* (52)) that employ PxpxPR motifs to target CIN85 and CD2AP, and thereby de-regulate host cell surface receptors to increase their chances of effective cell invasion.

### Regulation of phase separation in the cell

Liquid-liquid phase separation (LLPS) is a recently discovered, albeit fundamental molecular process that is driven by interactions between proteins and nucleic acids to form functionally specialised liquid compartments, the so called membraneless organelles, within cells (53). Phase-separated condensates enable the spatiotemporal segregation and increase the efficiency of cellular biochemical reactions due to selectively enriching the required macromolecules, while excluding others (54–56). Based on the main protein structural modules involved, most hitherto described LLPS systems belong to one of two major categories: (i) weak (often intrachain) residue-residue interactions between low-complexity, disordered regions of proteins (that might also interact with RNA) and (ii) SLiM-mediated interactions between disordered, motif-harbouring regions of proteins and the respective motif-binding domains in other proteins (53).

SLiM-mediated interactions typically contribute to receptor clustering and the formation of the associated, membrane-proximal signalling networks (so called signalosomes), such as the synaptic densities of excitatory (57,58) and inhibitory synapses (59), presynaptic active zones (60), T- and B-cell receptor signalosomes (61,62), the nephrin-associated signalling network specific for kidney podocytes (63), ABC transporter-linked condensates (64) and tip-link densities of stereocilia and microvilli of inner ear hair cells and intestinal enterocytes, respectively (65). SLiMs also play central roles in the assembly of several nuclear bodies, such as SPOP/DAXX bodies (66), Promyelocytic leukaemia nuclear bodies (67), nuclear splicing speckles (68), heterochromatin (69) and transcription regulatory condensates (70), as well as cytoplasmic phase-separated condensates of diverse functions, like yeast P-bodies (71), Balbiani bodies (72) and miRISC complexes (55), among others. When comprehensively collecting and classifying the SLiMs contributing to such processes, we were delighted to see that the majority of them belong to motif classes already present in the database, with most contributions coming from PDZ, SH3, SH2, FHA and PTB domain-binding motif classes.

Among the three novel motif classes functioning in LLPS processes, the LIG_DLG_GKlike_1 class collects phospholigands of the guanylate kinase-like (GK-like) domains of the discs large homologue (DLG) protein family that contains major scaffold proteins of postsynaptic densities (PSDs). The binding of PSD-95 to repeated phosphorylated motifs within SAP90 represents an essential link in the formation of PSDs (57,73).

PxpxPR motifs within the B-cell linker protein (BLNK, also called SLP-65) belong to the newly annotated LIG_SH3_CIN85_PxpxPR_1 motif class and mediate multivalent interactions with CIN85 trimers, forming the extended molecular scaffold underlying phase-separated B-cell receptor signalling clusters (62).

Another new class related to phase separation is found in algae belonging to the Chlorophyceae taxon that concentrate RuBisCO enzymes in a non-membranous compartment dedicated to CO_2_ fixation, called the Pyrenoid. Formation of the Pyrenoid matrix depends upon multivalent, low-affinity interactions between RuBisCO binding motifs (LIG_RuBisCO_WRxxL_1) of the protein EPYC1 and the RuBisCO small subunit (74,75).

### Pathogen hijacking

Due to their short lengths, SLiMs can evolve rapidly and hence are excellent conduits for convergent evolution and molecular mimicry. Several pathogens, including viruses, bacteria and eukaryotes are known to harbour protein sequences containing SLiMs used by their host organisms (6,7). These pathogenic SLiMs can contribute to interfacing with the host, rewiring normal cellular functions and hijacking processes for the pathogen's benefit. Several motif instances from pathogenic proteins have been already discussed, modulating the functions of the ESCRT system, RTK signalling, endocytosis and protein sorting. In addition, the current update of ELM describes several new SLiMs enabling pathogens to attach to and enter host cells and to subvert the cellular machinery to invade the host.

The SARS-CoV-2 virus, responsible for the COVID-19 pandemic, utilises SLiMs in two markedly different ways to enhance viral entry and infection: by the evolution of human SLiMs in viral proteins and the hijacking of SLiM-mediated interactions between human proteins. The Spike protein itself contains at least three SLiMs that are recognised by human proteins. It contains an RGD motif (LIG_Integrin_RGD_1), mediating interaction with integrins, a class of human cell surface receptors known to be targeted by several other viruses (76). In addition, the Spike protein also contains a multibasic cleavage motif that is recognised by human cell surface proteases (e.g. furin-like proteins of the PC protein family). The cleavage creates a new C-terminus in the Spike protein, which exhibits a third SLiM, CendR (LIG_NRP_CendR_1) recognised by neuropilin 1 (NRP1) (77). Blocking the Spike:NRP1 interaction was shown to restrict cell entry into NRP1-positive cells (9).

SARS-CoV-2 hijacks native human SLiM-mediated interactions inside the host cell as well, modulating the endocytic and autophagy machinery (8). These SLiMs are located in the disordered intracellular tails of the receptors targeted by Spike:ACE2 and various integrins, harbouring LC3-interacting region motifs (LIG_LIR_Gen_1) providing a direct molecular link to the autophagy machinery, as well as a phosphotyrosine domain**-**binding motif (LIG_PTB_Apo_2, LIG_PTB_Phospho_1), providing a direct interface to endocytosis components. In addition, ACE2 also contains SLiMs capable of interacting with I-BAR domains (LIG_IBAR_NPY_1), PDZ domains (LIG_PDZ_Class_1) an endocytic sorting signal (TRG_ENDOCYTIC_2), and SH2 domains (78). Most of the ACE2 and integrin motifs contain phosphosites, constituting molecular switches (79) regulating partner selection as well as binding affinity of the mediated interactions.

*Toxoplasma gondii* is an apicomplexan parasite that primarily infects cats and their prey but is also highly prevalent in the human population, being the causative agent of toxoplasmosis. All apicomplexans are intracellular parasites that invade host cells through the coordinated secretion of proteins contained in specialised organelles. Recent research has highlighted the role of SLiMs in host cell entry in Toxoplasma (52), in particular, the presence of the binding motifs for ALIX (LIG_LYPXL_S_1), TSG101 (LIG_PTAP_UEV_1, LIG_WW_1) and CIN85 (LIG_SH3_CIN85_PxpxPR_1) in Rhoptry proteins RON2, RON4 and RON5. These proteins, together with AMA1 and RON8, form the moving junction, an anchor point through which *T. gondii* introduces itself into the host cell, creating the parasitophorous vacuole. These motif instances provide a link to cytoskeletal rearrangement through members of the ESCRT system and microtubule-binding proteins (Figure 3).

## EXPLORING LINEAR MOTIFS

In addition to sharing the motif annotation data, the ELM server also enables exploratory analyses of SLiMs on the protein sequences submitted by the user. The search results are accessible via graphical and tabular representations with details of the regular expression matches. The graphical view provides an overlay of motif match information with the sequence's domain, secondary structure, and other key contextual features. Regions predicted by GlobPlot (80) to be in a disordered state are given a light green background, whereas for globular regions, where valid SLiMs are less likely, the background colour is pink. The summary table below the graphic provides total motif matches before and after applying logical filters based on contextual information. An example of setting up an ELM search and scrutinising the candidate motif matches is provided by using a viral protein, Human Cytomegalovirus UL135 (Figure 4).

While ELM provides a good starting point for motif exploration on a particular protein sequence, it is also expected that most regular expression matches are unlikely to be meaningful, especially for motif types that have low sequence complexity. Therefore, it is equally crucial to consider the biological context of the SLiM candidates. We have shared our recommendations to avoid typical mistakes during motif explorations (81). In our daily motif evaluation routine, we utilise specialised tools such as Jalview (82) and ProViz (83) to assess motif conservation in multiple sequence alignments, IUPred (84), DisProt (85) and MobiDB (86) to check the disorderliness and accessibility of the sequence regions with candidate SLiMs. To examine proteome-wide motif abundance, we query SLiMSearch (87) and ScanProsite (88). Inspection of the binding domain and its boundaries on the binding partner is done on Pfam (89), SMART (90) or InterPro (91).

Advances in experimental and computational methodologies continue to be added to the toolkit for SLiM investigations. Deep mutational scanning enabled systematic evaluation of the contribution for all residue possibilities within the LP cyclin motif (28). The cryo-EM ‘resolution revolution’ yielding structures of large macromolecular complexes is likely to be increasingly valuable in the future, especially for cases where the motif-binding interface involves more than one subunit on the binding partner, or when multiple motifs cooperatively bind to several subunits of a protein complex (92). Very recently, AlphaFold2 has been made available for protein structure prediction (93). AlphaFold's encouraging performance in protein structure prediction could potentially augment the interpretation and visualisation of SLiMs on the predicted protein models. However, at the time of publication, the confidence of IDR regions where SLiMs are enriched remains to be benchmarked for AlphaFold.

## CONCLUSIONS AND FUTURE PERSPECTIVES

The current update of the ELM database includes 30 novel SLiM classes, major revisions to five existing motif classes and a total of 411 new instances. New data developments in ELM have mainly focussed on cell cycle regulation, actin cytoskeleton regulation, membrane remodelling and sorting pathways, the formation of phase-separated liquid compartments and integrin signalling. These major areas of cell regulation are also heavily affected by pathogenic motif mimicry, therefore the current release of ELM not only captures information into how eukaryotic cells operate, but also into how they get efficiently invaded by various pathogens. In the coming years, the motif biology field and the ELM resource itself will need to tackle the challenge of integrating motif data as it becomes available from high-throughput motif discovery approaches (94–96), and the large volume of recently released and highly accurate protein structure prediction data (93) that undoubtedly hold the potential to revolutionise structural biology research.
